# Rare ectopic thyroid tissue as multiple bilateral pulmonary nodules: a case report and literature review

**DOI:** 10.1186/s13019-022-01962-z

**Published:** 2022-08-24

**Authors:** Jianfeng Tan, Jun Kuang, Yong Li, Ruian Wang, Shan Hu, Quanwei Guo, Jianhua Zhang

**Affiliations:** 1 Department of Thoracic Surgery, Shenzhen Hospital, Southern Medical University, No.1333 Xinhu Road, Baoan District, Shenzhen, 518101 Guangdong China; 2 Department of Nuclear Medicine, Shenzhen Hospital, Southern Medical University, No.1333 Xinhu Road, Baoan District, Shenzhen, 518101 Guangdong China; 3 Department of Pathology, Shenzhen Hospital, Southern Medical University, No.1333 Xinhu Road, Baoan District, Shenzhen, 518101 Guangdong China

**Keywords:** Ectopic thyroid, Pulmonary nodules, VATS, Case report

## Abstract

**Background:**

The prevalence of ectopic thyroid tissue is 1 in every 100,000 to 300,000 persons in the general population, and ectopic thyroid tissue in the bilateral lung lobes is even rarer. Due to its rarity, there is no definitive or standard guidance on the diagnosis and treatment of ectopic thyroid tissue presenting as multiple bilateral pulmonary nodules.

**Case presentation:**

A 56-year-old woman presented with multiple bilateral pulmonary nodules, and the patient had a history of hyperthyroidism but had no symptoms of ectopic thyroid tissue. Computed tomography (CT) demonstrated multiple solid nodules in both lungs, and the largest nodule (sized 15 × 14 mm) was located in segment 5 of the upper left lung. The initial diagnosis based on imaging was metastatic malignancies. Positron emission tomography-computed tomography (PET-CT) showed multiple bilateral intrapulmonary nodules that had slightly increased metabolism (SUVmax 1.7). The largest pulmonary nodule and another nodule in the left lung were resected by video-assisted thoracoscopy surgery (VATS). The pathological and immunohistochemical (IHC) examinations confirmed a diagnosis of ectopic thyroid tissue. No postoperative adjuvant therapy was given, and the patient was discharged 3 days after the operation and had regular follow-up examinations.

**Conclusion:**

The diagnosis of ectopic thyroid tissue in the bilateral lung lobes is extremely difficult and should be considered carefully. PET-CT and surgical resection of intrapulmonary nodules are alternatives for clinicians in diagnosing ectopic thyroid tissue. Regular postoperative follow-up is needed.

## Background

Ectopic thyroid tissue is a rare clinical condition with a prevalence of 1/100,000 to 300,000 individuals in the general population, and the incidence is much lower in men than in women [1]. Usually, aberrant ectopic thyroid tissue occurs along the midline and rarely in the gall bladder, lung, adrenal gland, heart or liver [2–6]. To our knowledge, most ectopic thyroid tissue in the lung is in a single lobe or homolateral lobes, and only 4 cases of ectopic intrapulmonary thyroid tissue in the bilateral lung lobes have been reported in the literature [7–10]. However, it is extremely difficult to diagnose multiple solid pulmonary nodules using only the patient’s clinical information and radiological findings. Positron emission tomography-computed tomography (PET-CT) and surgical resection should be recommended to determine the diagnosis. Here, we report a new case of ectopic thyroid tissue and discuss the current literature regarding the diagnosis and treatment of ectopic thyroid tissue in the bilateral lung lobes.

### Case presentation

**Fig. 1:**
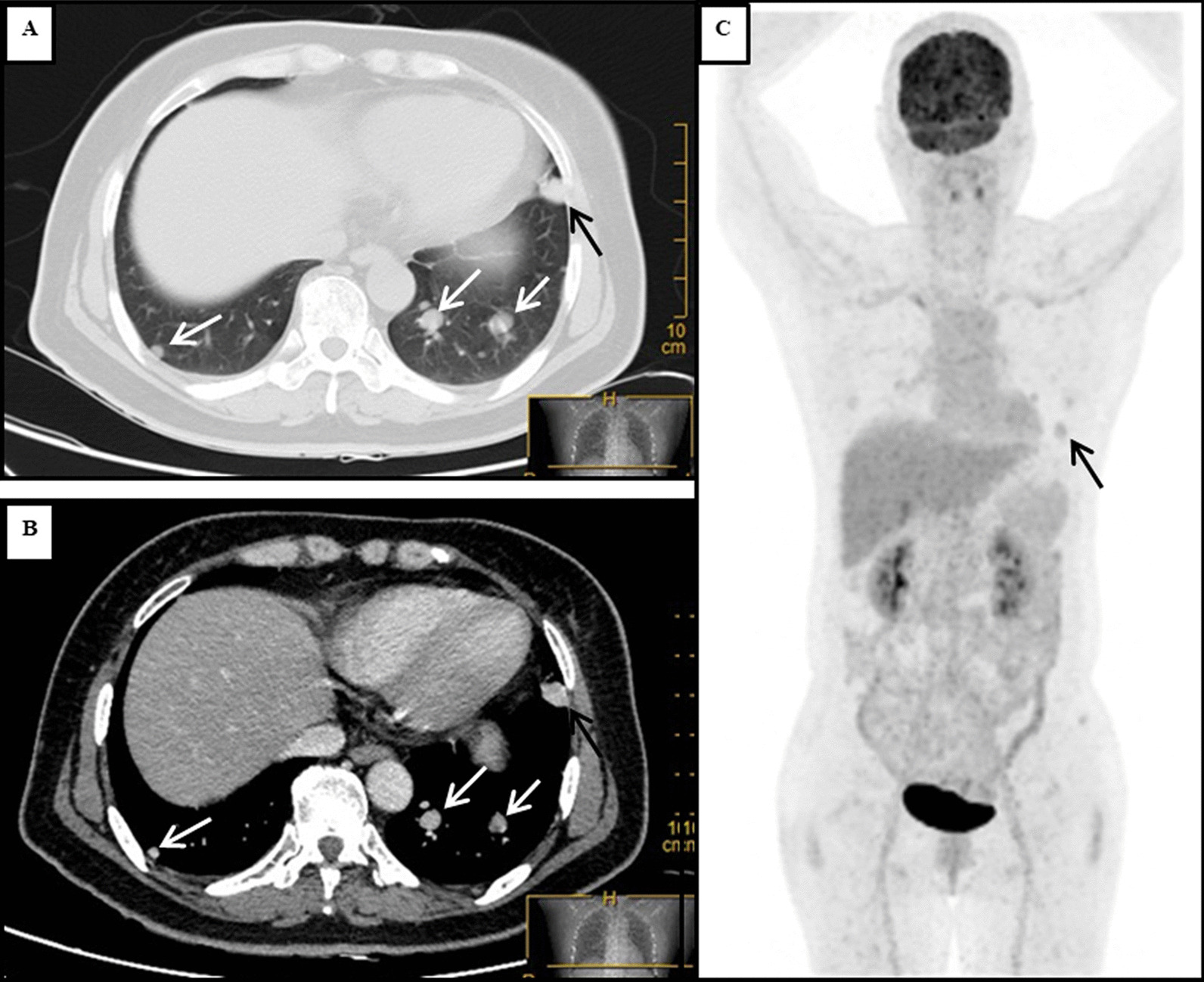
The chest CT scan and PET-CT examination showed multiple bilateral nodules in the patient’s lung lobes. The lung window (**A**) and longitudinal window (**B**) demonstrated that some round, well-defined and various-sized nodules were seen in both lungs (white arrow), the largest nodule was found in segment 5, and that nodule measured 15*14 mm (black arrow). (**C**) PET-CT indicated that there were multiple nodules in the bilateral lung lobes with mildly increased metabolism and that there was a larger nodule in the left lung (black arrow)

**Fig. 2:**
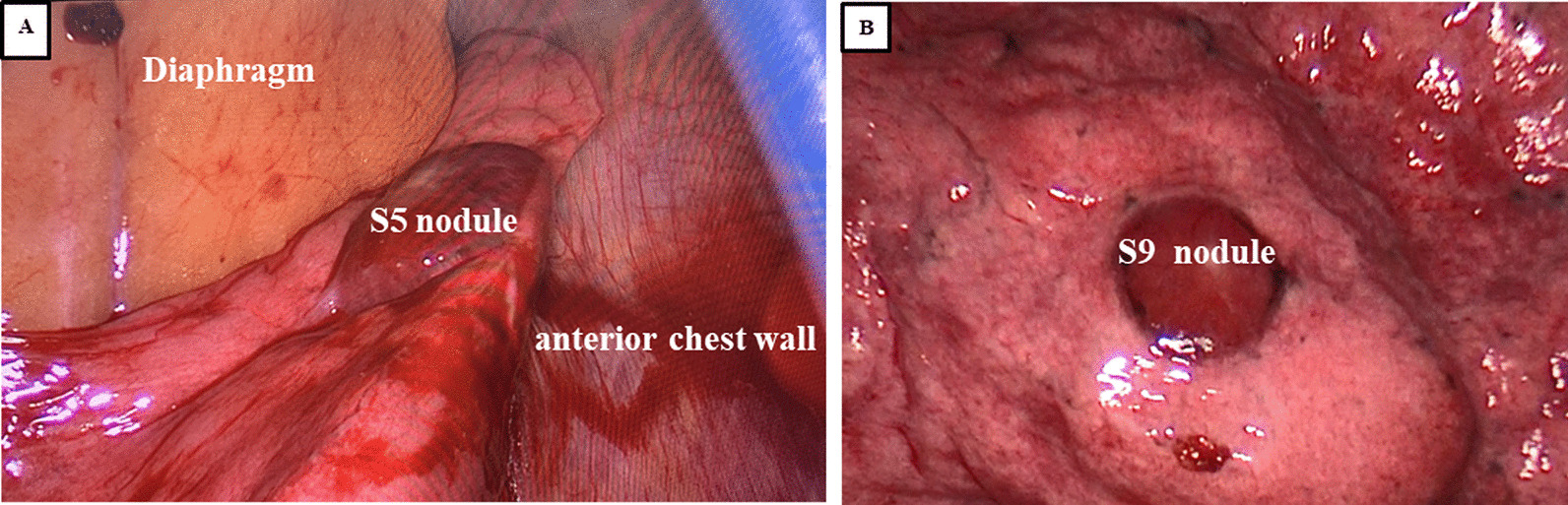
Intraoperative exploration revealed multiple nodules in the left lung that were dark red and soft, and these were protruding from the visceral pleura. The segment 5 (S5) nodule (**A**) and segment 9 (S9) nodule (**B**) were resected

**Fig. 3:**
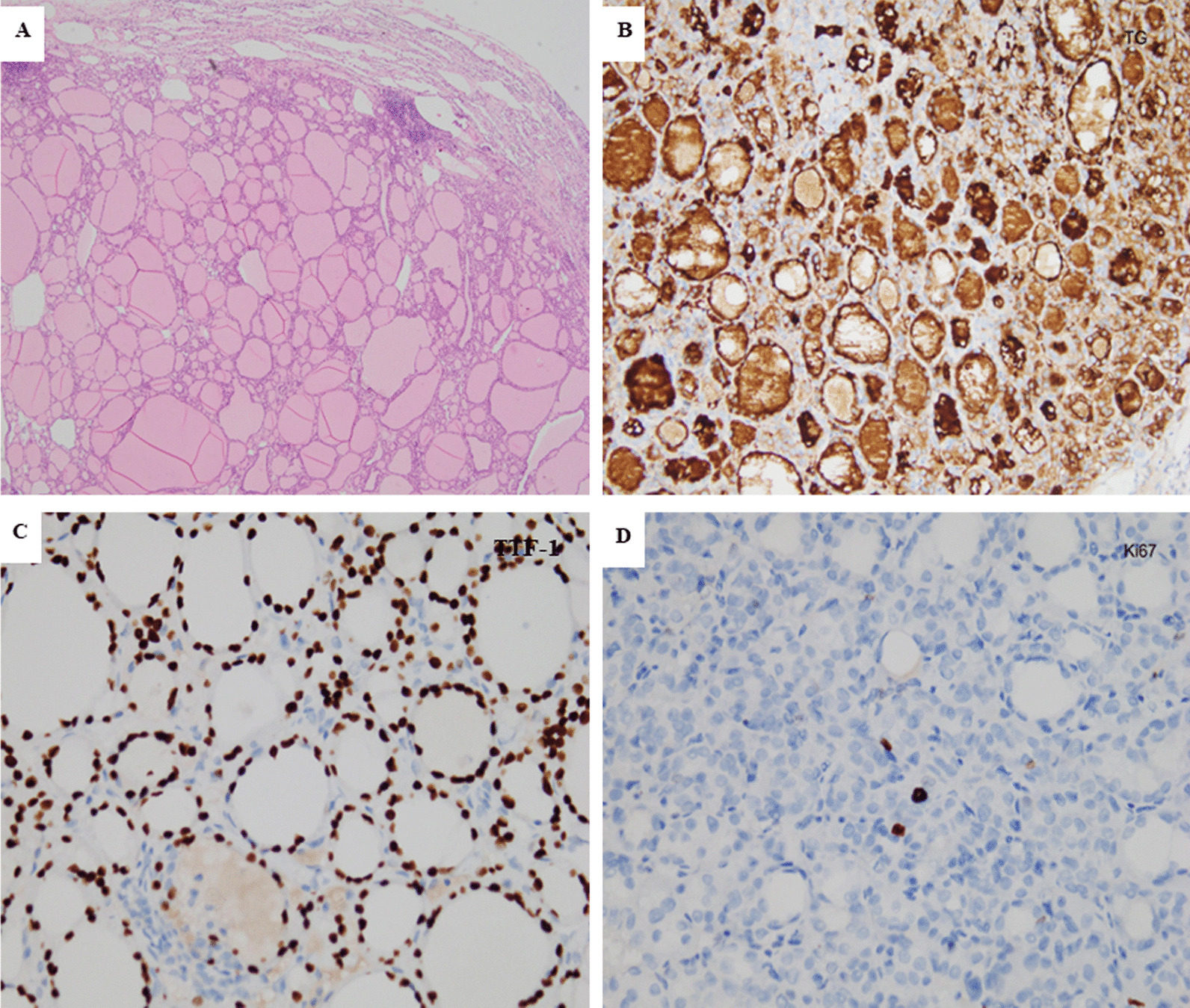
Microphotograph of the patient’s thyroid ectopia in the left lung lobes. **A** Haematoxylin and eosin (H&E) staining showed that the pulmonary nodules consisted of typical thyroid tissue with follicles containing colloids (magnification × 40). **B** Immunohistochemistry staining of the follicular epithelial cells showed positivity for thyroglobulin (TG, magnification ×100). **C** Positive immunohistochemical staining for thyroid transcription factor-1 (TTF-1, magnification ×100). **D** Immunohistochemistry staining of follicular epithelial cells showed weak positivity for Ki67 (< 1%, magnification × 100)

**Fig. 4:**
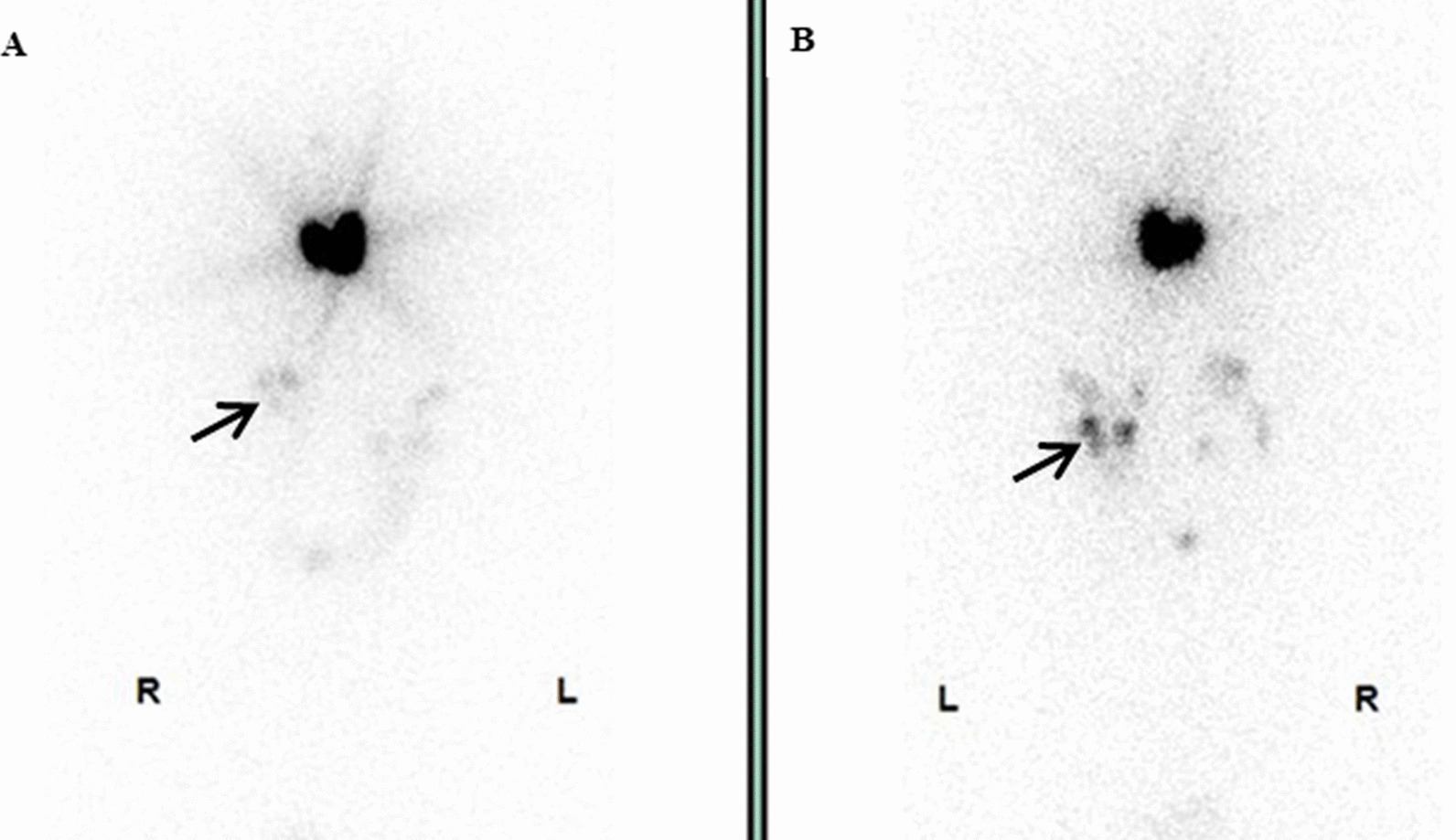
I-131 scintigraphy, anterior **A** and posterior **B** views showed that functional thyroid tissue had developed in the patient’s neck and that the multiple nodular radioactive distributions that were in the bilateral lung lobes were concentrated (black arrow)

A 56-year-old female patient was admitted to our hospital due to multiple bilateral pulmonary nodules on chest computed tomography (CT) during a routine medical examination.The patient underwent bilateral partial thyroidectomy for hyperthyroidism more than 20 years prior, and the patient’s thyroid function was normal after the operation. The patient had no previous history of alcoholism, smoking, tuberculosis, gynaecological or obstetric diseases, or malignant tumours, and no significant family history was found. The patient had no symptoms, such as cough, dyspnoea, fever, or weight loss. No obvious abnormalities were found on the patient’s physical examination. The patient’s thyroid function tests were normal, and her serum thyroid stimulating hormone (TSH) level was 1.96 μIU/mL (normal range, 0.27–4.20 μIU/mL), free triiodothyronine (FT3) level was 2.69 pg/mL (normal range, 2.00–4.40 pg/mL) and free thyroxine (FT4) level was 1.24 ng/dL (normal range, 0.93–1.70 ng/dL). Chest computed tomography (CT) showed the presence of multiple solid nodules in both lungs; the nodules were round, well-defined and various-sized, and the largest nodule (measured as 15*14 mm) was located in segment 5 of the upper left lung (Fig. 1A, B). Considering the patient’s radiological findings, we considered the diagnosis of pulmonary metastases despite a lack of evidence for malignancy. To further clarify the possibility of multiple lung metastases caused by occult malignant tumours, PET-CT was performed. The PET-CT results showed that the patient had multiple bilateral intrapulmonary nodules in her lung lobes, the nodules had slightly increased metabolism (SUVmax 1.7), and a larger nodule was found in the patient’s left lung (Fig. 1C). No metabolic abnormalities were found elsewhere in her body. Finally, a single-port thoracoscopic wedge resection was performed. Intraoperatively, we found that the largest pulmonary nodule was located in segment 5 (S5) of the left lung, and the nodule was dark red and soft and protruded from the visceral pleura (Fig. 2A). The pulmonary nodules in segment 5 (S5) and segment 9 (S9) were resected (Fig. 2B). The pathology results showed thyroid follicular tissue and pulmonary alveolar tissue without malignant cells (Fig. 3A). Immunohistochemical (IHC) staining indicated that the tissue was positive for thyroglobulin (TG) and thyroid transcription factor-1 (TTF-1) and that there was a strong reaction in the nucleus of the follicular cells (Fig. 3B, C). To exclude malignant neoplasms, IHC staining for Ki-67 was performed, and fewer than 1% of the cells were positive (Fig. 3D). I-131 scintigraphy suggested that functional thyroid tissue had developed in the patient’s neck and that the patient had multiple nodular radioactive distributions, which were concentrated in both of her lungs (Fig. 4), proving that the multiple intrapulmonary nodules had iodine uptake. Due to the lack of evidence of malignant tumours and after communicating with the patient and her family, the patient was discharged 3 days after the operation; to date, the patient has been followed up regularly for 1 year.

## Discussion and conclusions

Ectopic thyroid tissue in the bilateral lung lobes is extremely rare, with only 4 previously reported cases [7–10]. In the present case, we described a novel case of ectopic intrapulmonary thyroid tissue that occurred in both lungs, and this ectopic tissue was incidentally found on a medical screening examination. Due to the difficulty in diagnosing this patient’s condition, PET-CT and surgical resection were chosen to rule out occult malignancy. Ectopic thyroid tissue, which occurs due to developmental abnormalities during the migration of the thyroid anlage from the foramen caecum to its final position in the neck, is an unusual thyroid congenital abnormality [11]. Ectopic thyroid tissue can lead to different clinical symptoms, which can depend on the ectopic tissue’s location, size and state of malignancy. Often, most patients with ectopic thyroid tissue are asymptomatic, and even their thyroid function tests are normal, which makes the diagnosis difficult. In this case, the patient was asymptomatic with a history of hyperthyroidism, and her thyroid function tests indicated euthyroid. She was admitted to our hospital due to multiple bilateral pulmonary nodules on chest CT during a routine medical examination.Despite the lack of evidence of malignancy, we first considered that the patient’s multiple pulmonary nodules were likely to be metastatic malignancies.

Currently, identifying pulmonary nodules by surgical resection or invasive techniques is considered the most essential approach, and these diagnostic techniques can aid in determining the subsequent therapy [12]. Fine-needle aspiration cytology of lung nodules has been used to differentiate benign and malignant tumours. However, the sensitivity of fine-needle aspiration cytology in identifying the properties of nodules is approximately 90%, and there are certain diagnostic errors [13, 14]. Thoracoscopic resection and biopsy of pulmonary nodules can be used to not only explore the shape and nature of multiple pulmonary nodules but also to completely remove single or multiple pulmonary nodules and sample enough tissue to make a definitive diagnosis [15]. Because the accuracy of thoracoscopic biopsy is approximately 100%, thoracoscopic resection biopsy is more accurate than fine-needle aspiration cytology of lung nodules [16, 17]. In addition, single-port thoracoscopic resection and biopsy are very minimally invasive, with quick postoperative recovery and little effect on patients. After fully communicating with the patient and her family, we decided to explore and resect the largest pulmonary nodule in the left upper lung by single-port thoracoscopy. Surprisingly, the frozen section pathology results suggested normal thyroid tissue. We decided to resect the pulmonary nodule in segment 9 again because of the rarity of this condition, and frozen section pathology still indicated normal thyroid tissue. Postoperatively, the positive TG and TTF-1 results further supported ectopic thyroid tissue.

To date, there is no consensus or guidance on the diagnosis and treatment of ectopic thyroid tissue associated with multiple bilateral intrapulmonary nodules in clinical practice. Usually, ectopic thyroid patients have normal thyroid function and no symptoms, and these patients have no history of malignant tumours.[7, 10]. For ectopic thyroid patients with clinical symptoms or a history of malignancy, radioactive iodine ablation and surgical resection are the most common treatments [12]. However, the development of ectopic thyroid tissue into malignant tumours has also been reported [18]. Therefore, the diagnosis and treatment of ectopic thyroid cancer patients should be made more carefully, and metastatic cancer and primary thyroid cancer should be excluded by PET-CT first. Similarly, close follow-up of these patients is also very important. In our case, repeated re-examination showed that the patient’s thyroid function was normal and that there were no remarkable changes in the patient’s multiple bilateral pulmonary nodules.

Four cases of multiple bilateral nodules have previously been reported and diagnosed as ectopic goitre. Three patients were diagnosed with ectopic thyroid tissue without evidence of malignancy, and two of these patients were observed conservatively and were followed up regularly [7, 9, 10]. Another patient was diagnosed as having ectopic thyroid microfollicular adenoma by surgical resection [8].Interestingly, the four reported patients with bilateral pulmonary ectopic thyroid were from Asia: one Korean patient and three Chinese patients. Notably, two of the Chinese patients were from Wuhan, China. The fifth patient with bilateral ectopic thyroid reported in our case came from Shenzhen, China. Therefore, the incidence of bilateral ectopic thyroid may be related to race and region.In the case presented here, chest CT indicated that there were multiple bilateral nodules in the patient’s lung lobes and that there was a high-density rounded mass; PET-CT showed multiple intrapulmonary nodules with slightly increased metabolism, which corresponded with the features of ectopic thyroid tissue. Our case had a distinct difference compared with the previously reported cases, namely, that PET-CT was initially used to rule out malignant tumours and that surgical resection biopsy and I-131 were used to further confirm the diagnosis of ectopic thyroid tissue. After communicating with the patient and her family, she was discharged 3 days after the operation and chose regular follow-up.

In the present case, over 20 years had passed since the patient underwent bilateral partial thyroidectomy for hyperthyroidism, and pathology results showed benign lesions without malignant cells. We needed to determine the authenticity of the information provided by the patients and whether the cause of her hyperthyroidism was related to the intrapulmonary ectopic thyroid tissue. Re-examination of the previous surgical specimens from the thyroid glands was necessary. Unfortunately, those specimens could not be obtained because the patient underwent bilateral partial thyroidectomy in a remote county hospital 20 years prior, and the samples were no longer stored. This is the main limitation in the present case. In conclusion, we reported an exceedingly rare case of ectopic intrapulmonary thyroid tissue in the bilateral lung lobes. The diagnosis of ectopic thyroid is extremely difficult and should be cautiously considered.PET-CT and surgical resection of intrapulmonary nodules are alternatives for clinicians in diagnosing ectopic thyroid tissue. Regular postoperative follow-up is needed for these patients.

## Data Availability

The datasets used and/or analysed during the current study are available from the corresponding author on reasonable request.

## Abbreviations

TSH: Thyroid stimulating hormone
FT3: Free triiodothyronine
FT4: Free thyroxine
CT: Computed tomography
IHC: Immunohistochemical
TG: Thyroglobulin
TTF-1: Thyroid transcription factor-1
H&E: Haematoxylin and eosin
PET-CT: Positron emission tomography-computed tomography
